# Do interspecific hybrids lead to new evolutionary avenues in the plant family Lemnaceae?

**DOI:** 10.1111/nph.70904

**Published:** 2026-01-09

**Authors:** K. Sowjanya Sree, Klaus‐J. Appenroth

**Affiliations:** ^1^ School of Biotechnology, Institute of Science Banaras Hindu University Varanasi 221005 Uttar Pradesh India; ^2^ Matthias Schleiden Institute – Genetics I Friedrich Schiller University Jena 07743 Jena Germany

**Keywords:** duckweed, evolution, flowering, interspecific hybrids, *Lemna*, Lemnaceae, polyploids

## Abstract

This article is a Commentary on Lee *et al*. (2026), **250**: 629–647.

Hybridization in plants has been researched for a considerable amount of time and in several directions in terms of its role in evolution. However, some of the plants that predominantly propagate by vegetative means have not been in the limelight, in this context, probably due to the scarce availability of sexually propagating lab‐based bioresources. One such group of plants is the family Lemnaceae, which is commonly termed as duckweeds or water lentils. Although these are angiosperms capable of generative propagation, they majorly propagate by the budding of daughter fronds from mother fronds, that is by vegetative propagation (Landolt, 1986). The article published in this issue of *New Phytologist*, by Lee *et al*. (2026; pp. 629–647) entitled ‘Hybridity of mainly asexually propagating duckweeds in genus *Lemna* – dead end or breakthrough?’ has investigated inter‐ and intraspecific hybrids of different ploidy levels in two of the sections of the genus *Lemna*, *Alatae* and *Lemna*, of the family Lemnaceae, for their role in evolution of this group of aquatic monocots.

*Lee* et al. *found that all the allodiploid and allotriploid* L. × japonica *hybrids were infertile and did not produce any seeds*.

Plants of the family Lemnaceae are the smallest and fastest growing Angiosperms (Sree *et al*., 2015). They are aquatic monocotyledonous plants, ranging in size from *c*. 1 cm to less than a millimeter, that occupy lentic ecosystems. They are distributed world‐wide and are adapted to several different environmental conditions; however, some of the species have a narrow range of distribution and are endemic (Landolt, 1986). There is an increasing interest in their applications for wastewater treatment (Ziegler *et al*., 2017; Zhou *et al*., 2023), as a wholesome nutritional food source (Appenroth *et al*., 2017), and as a biofuel (Appenroth *et al*., 2021). For several aspects of these practical applications, knowledge of the genetic constitution and species identity of the duckweeds under study plays an important role.

Taxonomy of Lemnaceae has a long history of > 200 yr, starting with the definition of the family (Martinov, 1820), then morphological analyses and plant development (Hegelmaier, 1868) reaching a first threshold with the duckweed systematics research of the late E. Landolt (1986). Landolt's work on species delineation and developing a key for the determination of duckweed species was based on morphological and physiological investigations (Landolt, 1980; Bog *et al*., 2020). To the current‐day onlooker, it might read as a primitive method; however, owing to the small size of the whole plant (up to less than a millimeter), in addition to the extreme reduction in structural complexity, it was a herculean task. With the advancements in molecular biology, plastidic marker‐based delineation of duckweed species has been introduced, which has enabled more precise and reliable systematics of this plant family (Bog *et al*., 2019). A new era started with the use of the nucleus‐encoded tubulin genes for the molecular taxonomy of Lemnaceae and with the development of tubulin‐based polymorphism (TBP) fingerprinting of duckweeds (Braglia *et al*., 2021). This method, in contrast to plastid‐based analyses of DNA, made it possible to detect interspecific hybrids. As a first example, the species *Lemna japonica*, delineated based on morphological markers, was discovered to be a hybrid of the two species *Lemna minor* and *Lemna turionifera*, using this method. In a similar manner, an interspecific hybrid between *Lemna gibba* and *L. minor* was discovered and was called *Lemna* × *mediterranea* (Braglia *et al*., 2024), while *Lemna* × *aoukikusa* is an interspecific hybrid between *Lemna perpusilla* and *Lemna aequinoctialis*.

Detailed studies on inter‐ or intraspecific hybrids require a sexually propagating population of the parents of the hybrids, and if possible, also of the hybrids. Sexually propagating plants of duckweeds are not commonly available under laboratory conditions; hence, generative propagation of duckweeds has been hypothesized as a rare event in several publications. However, in order to confirm this, it is necessary to perform a significant number of ecological studies in the natural ecosystem of the concerned duckweed species. Such long‐term ecological investigations can be laborious and difficult, not only because of the field investigations involved and the presence of regular vegetative propagation in duckweeds but also because of the high probability of the frequency of epizoochory of duckweed plants from one water body to another, which could possibly change the population dynamics and interactions in a particular ecosystem. Lee *et al*. have circumvented the problems associated with obtaining naturally flowering plants by using salicylic acid or benzoic acid to induce flowering in laboratory‐grown duckweed cultures that were observed for floral development, pollen viability, seed set and seed germination. These cultures included plants belonging to two of the four sections of the genus *Lemna* viz., *Alatae* and *Lemna*, more precisely, 11 clones of the different species *L. minor*, *L. gibba*, *L. aequinoctialis, L. turionifera* and *L. perpusilla*, and 18 clones of different inter‐ and intraspecific hybrids. All clones were characterized by TBP, genomic *in situ* hybridization (GISH) and flow cytometry. This characterization has confirmed their species identity as well as the genetic status of these clones in terms of their ploidy.

The key point for evaluating the possible role of interspecific hybrids in evolution is the question of whether these hybrids are fertile. If not, these hybrids represent a dead end in evolution. Lee *et al*. found that all the allodiploid and allotriploid *L*. × *japonica* hybrids were infertile and did not produce any seeds. The same was the case with the hybrid, *L*. × *mediterranea*, as well as the triploid hybrids between *L. perpusilla* and *L. aequinoctialis*, but the tetraploid hybrid *L*. × *aoukikusa* was found to be fertile (Fig. 1). However, the infertile interspecific hybrids such as *L*. × *japonica* were able to propagate vegetatively. The formation of these triploid hybrids in the genus *Lemna* as investigated by Ernst *et al*. (2025) may be caused by the loss of triploid block. Interestingly, *L*. × *japonica* plants were collected from distant places across the globe (Lee *et al*., 2026). This brings us to the notion that the two parent species of *L*. × *japonica*, *L. minor* and *L. turionifera*, might flower regularly in nature and that the hybridization between these parent species might be a frequent event under natural conditions. Human or animal‐led introduction of these hybrid plants to distant places, as an alternative, is a far‐fetched idea. As pointed out previously, these assumptions need detailed ecological investigations, but from an evolutionary point of view, there will be no further progression in the direction of these infertile interspecific hybrids. Thus, interspecific hybrids in Lemnaceae, genus *Lemna,* can open a gate for evolutionary progress – but only in very rare cases. As per Lee *et al*., the section *Alatae* of the genus *Lemna* shows the potential for further speciation, thereby advancing the evolution of Lemnaceae.

**Fig. 1:**
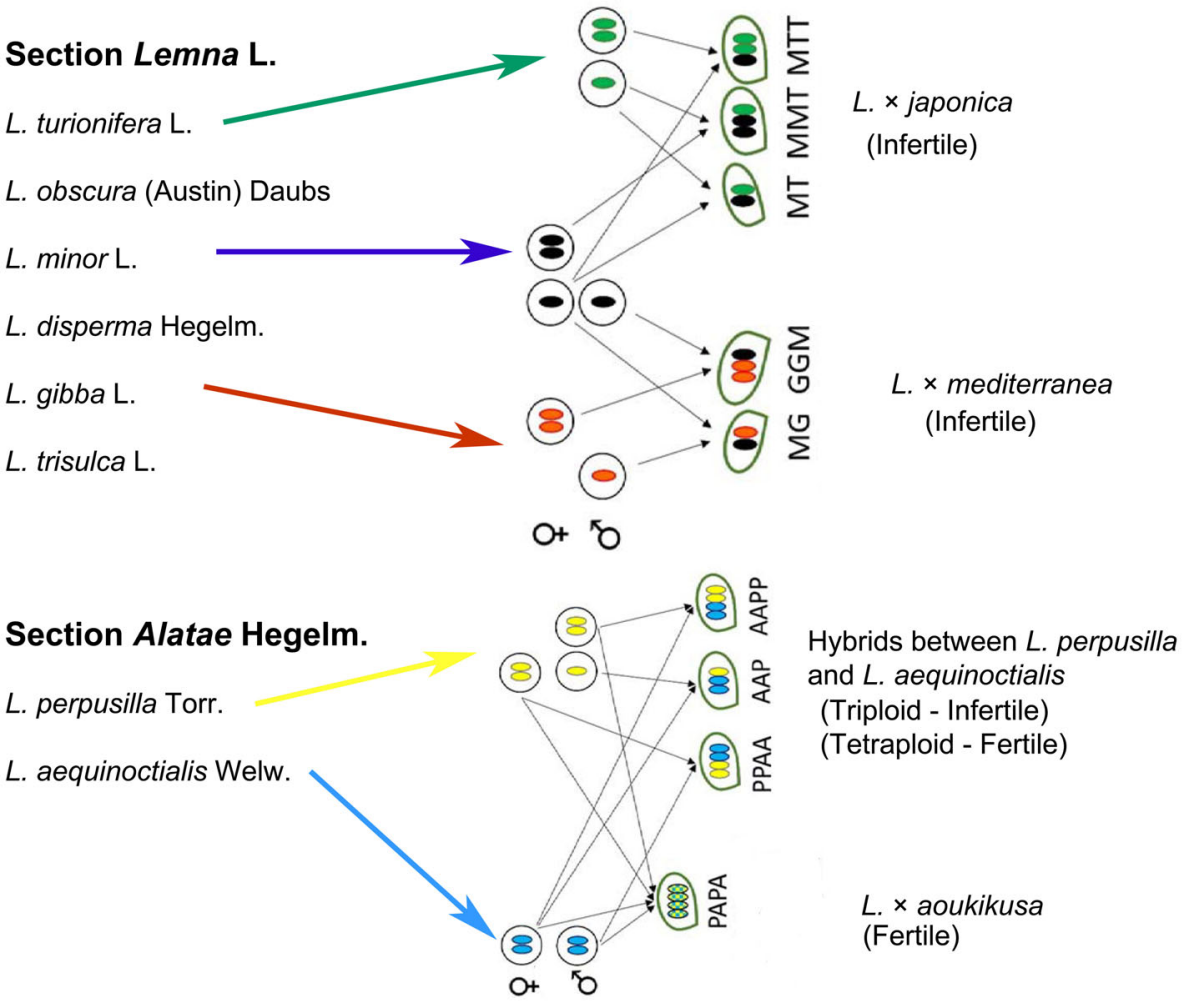
Interspecific hybrids in the sections *Lemna* and *Alatae* of the genus *Lemna* (Lemnaceae), and their female and male parents. Only gamete types involved in hybridization are shown. Figure adapted and modified from Lee *et al*. (2026; pp. 629–647, in this issue of *New Phytologist*). Upper case letters indicate the following genetic constitution: MG = *L. minor* × *L. gibba* (diploid); GGM = *L. gibba* × *L. minor* (triploid); MT = *L. minor* × *L. turionifera* (diploid); MTT = *L. minor* × *L. turionifera* (triploid); PAPA = *L. perpusilla* × *L. aequinoctialis* (tetraploid); PPAA = *L. perpusilla* × *L. aequinoctialis* (tetraploid); AAP = *L. aequinoctialis* × *L. perpusilla* (triploid); AAPP = *L. aequinoctialis* × *L. perpusilla* (tetraploid).

A future perspective could be to score for more of such hidden hybrids in the already morphologically delineated species. There are no indications of a similar situation in the genus *Spirodela*, comprising only two species, *S. polyrhiza* and *S. intermedia*, nor in the genus *Landoltia*, having only one species, *Landoltia punctata*. Promising candidates for similar investigations are the species belonging to the genera *Wolffiella* and *Wolffia*, which were delineated on a morphological basis and exhibit a high degree of reduction in structural complexity, making it extremely challenging for the use of morphological markers as taxonomic tools.

## Disclaimer

The New Phytologist Foundation remains neutral with regard to jurisdictional claims in maps and in any institutional affiliations.
